# Cell-lineage heterogeneity and driver mutation recurrence in pre-invasive breast neoplasia

**DOI:** 10.1186/s13073-015-0146-2

**Published:** 2015-04-09

**Authors:** Ziming Weng, Noah Spies, Shirley X Zhu, Daniel E Newburger, Dorna Kashef-Haghighi, Serafim Batzoglou, Arend Sidow, Robert B West

**Affiliations:** Department of Pathology, Stanford University School of Medicine, Stanford, CA 94305 USA; Department of Genetics, Stanford University School of Medicine, Stanford, CA 94305 USA; Biomedical Informatics Training Program, Stanford University, Stanford, CA 94305 USA; Department of Computer Science, Stanford University, Stanford, CA 94305 USA

## Abstract

**Background:**

All cells in an individual are related to one another by a bifurcating lineage tree, in which each node is an ancestral cell that divided into two, each branch connects two nodes, and the root is the zygote. When a somatic mutation occurs in an ancestral cell, all its descendants carry the mutation, which can then serve as a lineage marker for the phylogenetic reconstruction of tumor progression. Using this concept, we investigate cell lineage relationships and genetic heterogeneity of pre-invasive neoplasias compared to invasive carcinomas.

**Methods:**

We deeply sequenced over a thousand phylogenetically informative somatic variants in 66 morphologically independent samples from six patients that represent a spectrum of normal, early neoplasia, carcinoma *in situ*, and invasive carcinoma. For each patient, we obtained a highly resolved lineage tree that establishes the phylogenetic relationships among the pre-invasive lesions and with the invasive carcinoma.

**Results:**

The trees reveal lineage heterogeneity of pre-invasive lesions, both within the same lesion, and between histologically similar ones. On the basis of the lineage trees, we identified a large number of independent recurrences of PIK3CA H1047 mutations in separate lesions in four of the six patients, often separate from the diagnostic carcinoma.

**Conclusions:**

Our analyses demonstrate that multi-sample phylogenetic inference provides insights on the origin of driver mutations, lineage heterogeneity of neoplastic proliferations, and the relationship of genomically aberrant neoplasias with the primary tumors. PIK3CA driver mutations may be comparatively benign inducers of cellular proliferation.

**Electronic supplementary material:**

The online version of this article (doi:10.1186/s13073-015-0146-2) contains supplementary material, which is available to authorized users.

## Background

Cancer evolution is driven by the accumulation of somatic mutations that confer fitness advantages to the tumor cells, enabling them to evade homeostatic mechanisms and to proliferate. Leveraging advancements in next-generation sequencing technologies, large-scale efforts have cataloged the somatic mutational events driving the progression of cancer [1-3]. These efforts, as well as studies sampling multiple different locations in the same tumor [4-6], have revealed that the tumor mass itself is genetically heterogeneous [7]. At a single cell level, studies reveal substantial tumor heterogeneity [8].

To understand the foundations of tumor heterogeneity, and the dynamics of cell growth in the tumor and related proliferative lesions, it is important to recognize that all cells in our somas, including those of cancers, are related to one another by a bifurcating lineage tree whose root is the zygote. In that tree, each node is an ancestral cell that divided into two, and each branch connecting two nodes represents at least one cell cycle (though usually many more than one). When a somatic mutation occurs in an ancestral cell, all its descendants carry the mutation, which can then serve as a lineage marker if assayed in multiple samples from the same individual. Phylogenetic reconstruction of tumor progression, with the goal of deriving an evolutionary tree of the tumor and related tissue samples, can then be performed on the basis of the presence of the somatic mutations across the diversity of samples from that patient.

Most genetic and epigenetic studies of clonal heterogeneity have focused on the primary invasive and metastatic stages [9-13]. By contrast, there have been few genetic analyses of significant depth that examine the spectrum of the pre-invasive compartment, represented by lesions ranging from hyperplasias to carcinoma *in situ*. The pre-invasive compartment is becoming increasingly clinically significant as cancer screening programs have led to a tremendous rise in the diagnosis of lesions in this category [14,15]. Noninvasive neoplastic proliferations of breast tissue, including hyperplasias and columnar cell lesions, represent a range of phenotypic intermediates, and are associated with mildly increased risk of carcinoma. In a prior study we established that a subset of these neoplasms were closely related to the concurrent invasive carcinoma [16]; those neoplasms carried a significant mutational burden and contained characteristic genomic alterations such as chromosome amplifications. Other lesions arose from much earlier ancestral cells and did not exhibit a large mutational burden, nor chromosome alterations.

For a better understanding of genetic heterogeneity of pre-invasive lesions we performed targeted deep sequencing of multiple samples from six breast cancer patients. We characterized the evolution of the patients’ neoplastic lesions and their phylogenetic relationships with the diagnostic carcinoma. Building on a prior study in which we deeply sequenced 31 somatic genomes of tumors, neoplasias, and controls in six patients [16], we here obtain much more accurate estimates of variant allele frequencies (VAFs) of phylogenetically informative single nucleotide variants (SNVs) in a much greater number of samples, with emphasis on pre-invasive neoplasias. In particular, we expanded the number of samples to 66, including 18 carcinomas and 34 concurrent neoplasias. From more than a thousand phylogenetically informative SNVs (on average 181 per patient), we reconstructed highly resolved trees that illuminate the history of clonal expansion and cell-lineage heterogeneity of these cases, as well as the occurrence of independent PIK3CA mutations within the same patient.

## Methods

### Selection of archive neoplasias

The study was conducted under a waiver by the Stanford IRB.

Six breast cancer patients with concurrent neoplastic lesions (ranging from early neoplasia, carcinoma *in situ*, and invasive carcinoma, as well as normal control) were selected for a previous study [16]. In this study, we added 38 mostly pre-invasive lesions and deeply sequenced all lesions (including the previous ones except three of them in patient 1) for 1,185 PCR-targeted SNVs (Additional file 1).

### DNA isolation and library preparation

DNA was extracted from each sample using protocols optimized for archival material. For samples of the discovery set [16] we started with de-paraffinization of tissue sections with xylene (50°C and two to three repeats can increase the DNA yield), then cell lysis with Proteinase K (overnight at 50 to 56°C), followed by column-based DNA purification Life Technologies, catalog number AM1975 (Carlsbad, CA, US), or Qiagen, catalog number 56404 (Venlo, Limburg, US).

For the new samples of this study for which little tissue (a single small core) was available, we performed the same de-paraffinization and cell lysis as above. To maximize the DNA recovery, we then removed proteins and other cell debris by two precipitations. The first precipitation was performed with Protein Precipitation Solution (Qiagen, catalog number 8241679) to remove protein and cell debris. The second precipitation was performed with isopropanol Sigma, catalog number 19516 (St. Louis, MO, US)) and glycogen to pellet all DNA in the supernatant. After being completely dissolved in 50 μl TE, DNA was further purified with a Qiagen MinElute column (catalog number 28004) and eluted in 30 μl EB buffer.

We used either 10 ng genomic DNA purified from each original sample or 2 ng DNA of each new sample. For each patient, a random subset of SNVs from each phylogenetic class from the discovery set, as well as the SNVs of PIK3CA, were chosen for targeting. Libraries for targeted sequencing were generated by multiplexed PCR with primers containing Illumina adaptor sequences. The multiple target-specific regions from each sample were amplified by an initial PCR (98°C/1 minute; 25 cycles of 98°C/10 s, 72°C/10 s, 65°C/1 minute, 60°C/8 minutes, 65°C/3 minutes, and 72°C/30 s; 72°C/5 minutes; hold at 4°C) with 50 nM of combined multiplex primers. PCR products of the target size were isolated by gel purification. A unique barcode was then added to each sample by a second PCR using Illumina-compatible primers. All barcoded samples from two or more patients were pooled and sequenced with a single lane of HiSeq2000 to obtain high sequence coverage of the targeted SNVs for the accurate estimation of their VAFs.

### Primer design and variant allele frequency quantification

A custom pipeline for primer design was developed to accommodate a multiplex PCR amplification strategy that directly incorporates Illumina-compatible primers for subsequent sequencing. Primers were designed in a way such that 1) size of the target PCR amplicon was about 100 bp, 2) the target SNV was within approximately 40 bases of the sequence start site, and 3) all primers for the same patient were compatible for multiplexing.

To generate primers for a given target SNV, Primer3 software [17,18] was used to produce sequences for up to 50 primer pairs with the following characteristics: 18 to 24 bp primer length, 100 to 160 bp length product size, and a minimum of 4 bp distance between the mutation site and either primer. Subsequent filtering steps removed any primers that violated the following constraints: maximum of 50 bp distance between the mutation and one of the primers in a pair, unique gapless alignment of both primers in a pair (as single reads) with tolerance of up to one mismatch, unique alignment of primer pairs via paired alignment with Bowtie 0.12.7 [19]. Illumina-compatible adapters were then added to each primer pair. In a final step, a single primer pair was selected for each locus using a greedy algorithm that iteratively reduced cross-dimerization scores among primers for all loci intended for multiplex processing.

Reads were mapped to the human genome assembly hg19 using the DNAnexus read mapper. Reads overlapping each SNV position were extracted from the mapped BAM files and the numbers of reads supporting the reference or alternative allele were counted. Because multiple oncogenic mutations have previously been reported at PIK3CA HIS1047, reads supporting all four nucleotides were counted at that position.

### Single nucleotide variant statistics

Of the 1,185 SNVs that were tested, 7 gave fewer than a total of 1,000 reads, which we denote as PCR failures. Fifty-five SNVs turned out to have been false-positive calls in the original study [16], a high rate because we biased the selection of SNVs to be tested in this study towards potentially interesting or ambiguous SNVs. Sixteen were germline SNVs. Among 1,107 validated SNVs, 19 are in ambiguous phylogenetic classes. Thus, a total of 1,088 SNVs or 11,474 data points were available for treebuilding.

### Treebuilding

Inspection of all 12,445 data points (the sum of all patients’ SNVs across all samples, excluding PCR failures) revealed surprisingly clear separation between negatives and positives (Additional file 2). We used a VAF of 0.02 as the initial default cutoff to separate negatives from positives. This allowed construction of a simple presence-absence vector for each SNV with one value (1 or 0) for each sample in that patient. We here refer to the presence-absence vector as the 'phylogenetic pattern' or simply 'pattern'.

Our strategy for treebuilding, manually executed in Excel with repeated detailed examinations of all data points, was to first identify the robust phylogenetic classes. These consist of patterns that are supported by more than one SNV, and whose SNV VAF values across samples are consistent with each other. This was intended to avoid overfitting the tree on the basis of noisy SNVs, which would be especially misleading when untrue mixed-lineage samples are inferred. In rare instances (N = 4) we therefore allowed a single SNV to constitute a robust class if the VAFs were clearly positive and its inclusion would not generate an additional mixed-lineage sample. Three SNVs had VAFs in specific samples that were below 0.02 but were considered positive because all other values matched that class well; similarly, nine SNVs had VAFs in specific samples that were above 0.02 but were considered negative (Additional file 2). Including or excluding these SNVs did not affect the trees.

We inferred the trees from the patterns with straight-forward parsimony, with one simple modification that would allow for the presence of mixed-lineage samples. Phylogenetic patterns that ‘conflict’ in that they cause a sample to not be unambiguously placed induce a 'duplication' of the sample such that the two copies can be placed independently in the tree and are then joined for display and interpretation. Five samples of the 66 total were inferred to be derived from two lineages. No phylogenetic patterns conflicted with each other beyond these. The contribution of VAFs from each mixed lineage summed to match the average VAF of the ancestral lineage, consistent with the presence of the mixed lineages.

### Data access

The raw fastq sequence data from this study have been submitted to NCBI [20] under BioProject identifier PRJNA193652.

## Results

### Targeted deep sequencing of somatic SNVs in multiple neoplastic samples from each patient

The assayed lesions included the morphologic categories of breast neoplasias (apocrine metaplasia, usual ductal hyperplasia, and columnar cell lesion (CCL)), hyperplasias with atypia ('AH', atypical ductal hyperplasia and 'FEA', flat epithelial atypia), ductal carcinoma *in situ* (DCIS), invasive ductal carcinoma (IDC), and metastasis. From the original 12,383 somatic SNVs of the six patients, we picked 1,185 (ranging from 130 to 296 per patient) for deep targeted resequencing in order to obtain highly accurate VAFs for building high-resolution lineage trees of all samples from each patient. We randomly selected subsets of SNVs from each branch of the original trees to maximize the diversity of phylogenetic patterns and therefore the expected resolution of the expanded trees. In addition, we included SNVs that violated the original trees, such as germline SNPs with dropouts in some samples, genuine somatic SNVs that mark alternative lineages, and recurrent SNVs that arise independently multiple times in the same patient.

We designed, for each patient, a fully multiplexed targeted PCR assay with each sample having a unique barcode, and sequenced the products with the Illumina HiSeq platform. The number of reads per patient ranged from >60 million to >283 million and the resulting deep coverage allowed highly accurate VAF estimates (Additional files 1 and 3). Background false positive rates estimated from the large number of SNVs absent from subsets of samples were well below 1 in 1,000, consistent with the rates of Illumina sequencing errors. We found no evidence for PCR jackpots in the distribution of sequencing errors, suggesting that the sequencing libraries were prepared with sufficient starting material to avoid these types of effects.

**Figure 1:**
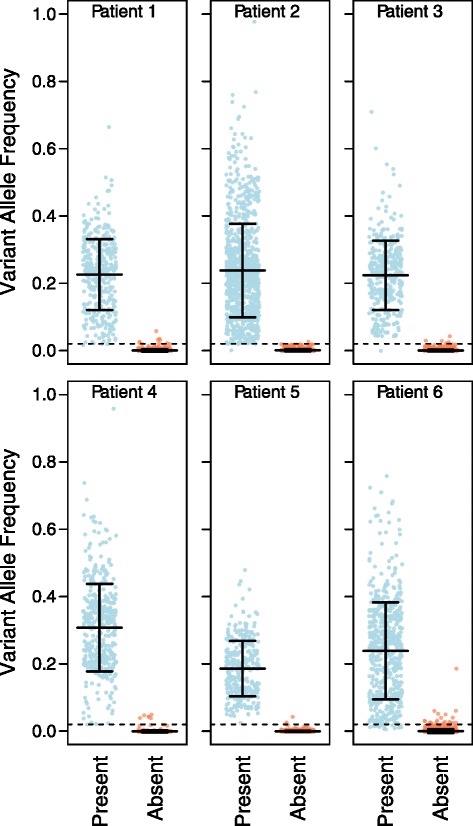
**Variant allele frequencies from the six patients.** Each SNV by sample combination is represented by a point, colored blue if it was called present in a sample, or orange if it was called absent. The 0.02 VAF cutoff used to determine presence or absence for most variants is shown as a dashed horizontal line. Means and standard deviations are shown, which are so close in the absent classes that they appear as a single horizontal line.

Separate tabulation of these data for each patient resulted in a matrix of VAFs for each SNV (rows) in each sample (columns) (Additional file 3). Of the 1,185 amplicons, 7 did not generate sufficient numbers of reads (N < 1,000; PCR failures) and were excluded from subsequent analysis (Additional file 1). For each patient, each VAF matrix was converted to a presence/absence matrix as previously described [16], where samples were called positive for an SNV’s alternative allele if its frequency was 0.02 or greater (Figure 1). Of the 1,178 PCR positive assays, 16 had alternative alleles that were present in all samples of the patient. These represent germline SNVs that were false negatives in one or more samples in the original study. Fifty-five assays did not have an alternative allele, representing false positives in the original study. Most of these false negatives and false positives are from groups that violate the original tree. Thus, 1,107 variants (93.4%) were *bona fide* somatic SNVs. SNVs exhibiting similar patterns of presence/absence among samples were grouped together into phylogenetic classes. Nineteen SNVs (1.7%) were categorized as belonging to ambiguous phylogenetic classes. The remaining 1,088 SNVs were categorized as belonging to unambiguous classes from which trees were built.

### Ancestral cell relationships revealed by highly resolved phylogenetic trees

**Figure 2:**
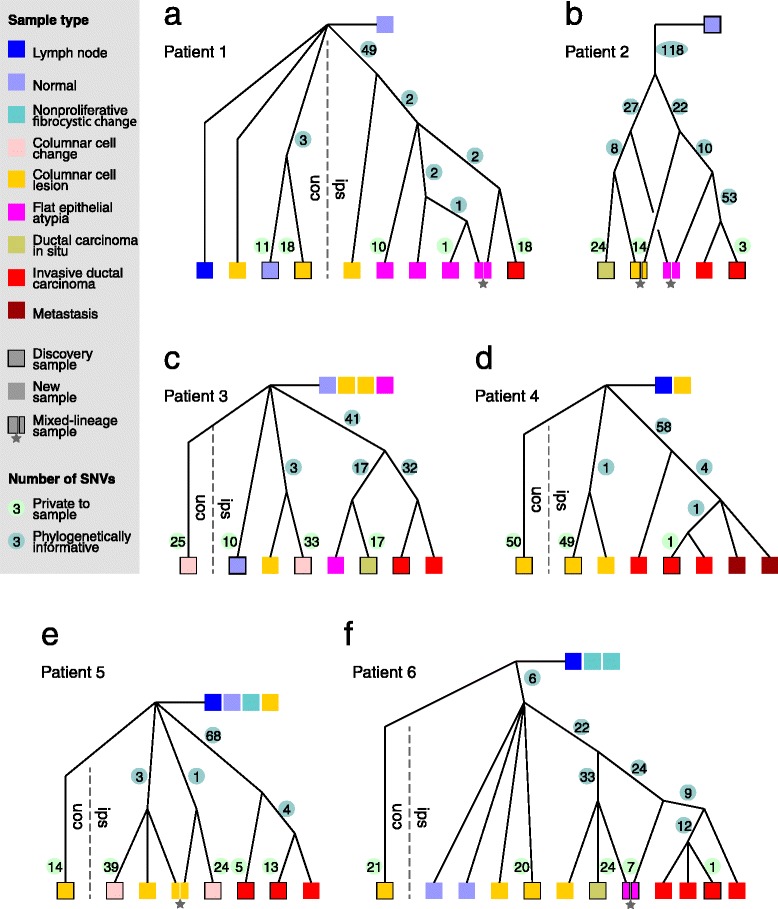
**Lineage trees inferred from 1,088 SNVs from the six patients.** Numbers in circles are the number of SNVs that arose in the branch leading to an ancestral node (blue) or that are private to the sample (green). Con, contralateral; Ips, ipsilateral. Stars denote samples originating from at least two lineages. For these samples, the estimated proportions of each lineage’s contribution is denoted by the sizes of the samples’ rectangles. Samples whose branches are drawn rightward at the top of the tree do not harbor any of the assayed SNVs.

Applying parsimonious inference on the phylogenetically informative classes, we obtained one tree per patient (Figure 2) that is fully consistent and without conflicts (with two SNVs being exceptions, discussed below). The resolution of the branching order is high, with all trees having mostly bifurcations (indicating unambiguous branching) and only a few tri- and multifurcations (indicating uncertainty in branching order). The multifurcations are concentrated at the tops of the trees, where there are no mutations to resolve branching order and where the samples that do not have any of the assayed somatic variants branch off the root node. In all five patients for whom we have contralateral samples, the left-right cell division occurring in early embryonic development appears near the root of the tree, with few mutations able to distinguish between the contralateral and ipsilateral lineages. In contrast, tumor evolution, characterized by a larger number of cell divisions and higher mutation rates, produces greater resolution in branching order towards the leaves of the trees [21]. The trees from the discovery set (from our original study, which was based on whole-genome data with lower coverage and therefore less confident VAF estimates [16]) are exactly represented as strongly supported subtrees, highlighting the reproducibility and robustness of the data and the fidelity of somatic mutations as lineage markers.

Most (N = 20) pre-invasive neoplastic lesions whose histological characteristics are closest to that of normal epithelial cells either have no common ancestors with one another and branch off the root node, or have shared common ancestors with other samples high in the tree. Only one of these assayed neoplasias (in patient 2) has an aberrant genome [16] and a genetic relationship with the concurrent carcinomas (Figure 2b). In the four patients (patients 1, 2, 3, and 6) that had DCIS or AH lesions, more recent ancestral cell divisions invariably produced one daughter cell that would go on to form the IDC and one that would produce the DCIS and/or AH (Figure 2a-c,f). Based on their relationships with DCIS and IDC, AHs represent an advanced form of pre-invasive lesion with a greater mutation burden than the typical ductal hyperplasia or CCL.

### Phylogenetic heterogeneity within pre-invasive samples

In contrast to trees produced from single cells or organismal phylogenetics, evolutionary inference on tumor samples that contain many cells is complicated by the potential presence of mixed-lineage samples. To identify such phylogenetic heterogeneity, we identified SNV classes whose presence/absence patterns cannot be explained by a single tree. None of the DCISs or IDCs came from a mixed lineage. This does not apply to the potential presence of subclones within the IDC samples, for which this study was not designed. Rather, our data indicate that no DCISs or IDCs were composed of two independent ancestral cell lineages, but that a substantial majority of the material of all carcinomas originated from a single ancestral cell. We cannot identify small contributions of independent lineages, but the VAF distributions in the IDCs prove them to be minor if they exist at all. By contrast, five non-carcinoma samples from four patients (1, 2, 5, and 6; two CCLs and three AHs) exhibited mixed-lineage origins (Figure 2a-b,e-f). Each is defined by two classes of SNVs whose presence/absence patterns cannot be explained by a single tree. Instead, after a period of common ancestry that is represented by ancestral SNVs present at high frequency, there was a cell division that gave rise to two independent lineages, each with its own unique subtree, which accumulated unique SNVs that distinguish the lineages from each other. Progeny from those lineages proliferated in close proximity and with similar phenotypes, eventually contributing almost equal numbers of cells to the samples. Because the lineage-specific SNVs are present in only about half of the lesional cells each, in mutually exclusive subsets, their VAFs are about half of the VAFs of ancestral SNVs carried by all cells of the sample (Additional file 3), providing further confirmation of their presence.

### Within-patient recurrences of PIK3CA driver mutations

The only previously identified gene-specific driver changes in our patients were the HER2 amplifications in patient 6 and two missense mutations in PIK3CA in patients 1, 3, 4, and 5. Our highly refined trees provide an opportunity to ask about their origins relative to the cellular ancestors that our trees have identified. In phylogenetic terms, we asked in which branches of the tree, and thereby how early or how late, these changes occurred. In the only HER2-positive patient (patient 6) of our study, a roughly 4 MB amplification including the *HER2* gene occurred in a lineage ancestral to the last common ancestor of the DCIS, FEA, and IDCs (Figure 2f, in the branch with 22 SNVs). On that same branch, the majority of the aneuploidies in this patient arose, followed by some additional aneuploidies and point mutations in the ancestral lineage of the IDC [16].

**Figure 3:**
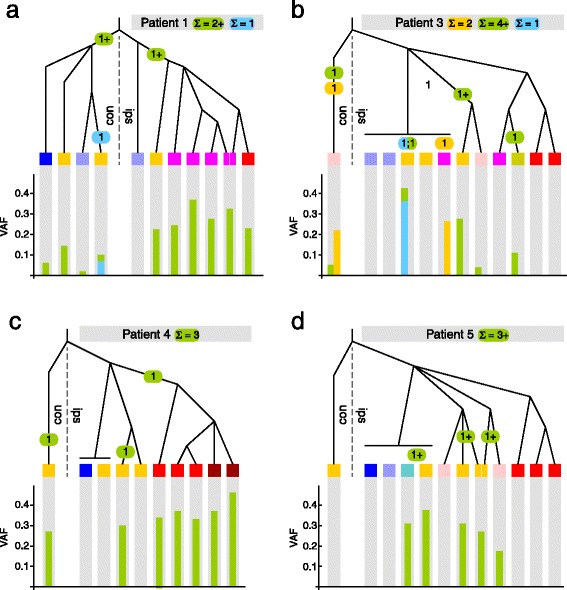
**Inferred origins of recurrent mutations and their VAFs.** Sample color coding is as in Figure 2. Green bars, VAF of PIK3CA H1047R; blue bars, VAF of PIK3CA H1047L. Green or blue circles on branches mark the origin of the indicated number of mutations, where a plus sign indicates that there may be additional events that we cannot resolve. Orange bars and circles pertain to the T-to-C change in position 101,160,839 on chromosome 11 in patient 3.

The single origin of the HER2 amplification in patient 6 is in stark contrast to position 178,952,085 on chromosome 3, which encodes amino acid 1,047 in PIK3CA. An A-to-G mutation, which causes the H1047R missense change, is present in four patients (1, 3, 4, and 5; Figure 3). In addition, two of these patients also carry an A-to-T change (H1047L). Consistent with previously reported frequencies [22-25] these patients harbor this known driver mutation at high VAFs that are in the range of most other phylogenetically informative variants (Figure 3). However, in each of the patients its presence/absence pattern across the samples indicated multiple independent origins, given the tree topologies. In patients 1, 3, and 4 it arose independently both contralaterally and ipsilaterally. Ipsilaterally, where the trees are highly resolved due to the larger number of samples, H1047R arose at least once in patient 1, at least three times in patient 3, twice in patient 4, and at least three times in patient 5. In addition to H1047R, H1047L occurred once in patient 1 and once in patient 3, in both instances in a neoplasia that also carries H1047R (Figure 3).

## Discussion

Pre-invasive neoplastic lesions are increasingly recognized as being important clinically (in diagnostics) and scientifically (for mechanistic insight into cancer evolution). We address both aspects by performing phylogenetic inference with somatic SNVs across the morphologic spectrum of neoplastic progression from normal breast epithelium to invasive breast cancer. We deep-sequenced 1,107 somatic SNVs in 66 samples from six patients and reconstructed the single most parsimonious evolutionary tree for each patient. The trees reveal insights into three aspects of cancer evolution that are of future clinical relevance: phylogenetic relationships among the pre-invasive lesions and with the IDCs, lineage heterogeneity of pre-invasive lesions, and recurrence of mutations in PIK3CA.

### Phylogenetic relationships of neoplasias

Our trees cast the traditional concept of tumor progression into a new light, in which the idea of ‘early’ neoplastic lesions ‘progressing’ to a tumor phenotype is refined on the basis of cell lineage inference. Pre-invasive neoplastic lesions are not the direct precursors of invasive lesions but independent clonal proliferations of an ancestral cell (or in the case of mixed-lineage samples, a few ancestral cells), just as the carcinomas are. By tracing the lineage from the root of each tree to the IDC ancestor, a progression of the cellular lineage from mildly to strongly proliferative and eventually invasive phenotypes can be inferred.

The trees prove it to be a single cell lineage and not a field of cells that ‘progress’. The single-cell lineage concept is analogous to the germline, where many cells proliferate into germ cells but only one eventually grows into the organism. Here too, there is cell proliferation during lineage evolution, but the ancestral nodes represent single cells revealed by clonal expansion of their progeny. The ancestral lineages represent many cell divisions, but the other daughter cells of these divisions did not independently proliferate into visible lesions, or else the tissue would have been a massive collection of pre-invasive lesional material that would have been detected much before formation of the diagnostic IDC.

The trees reveal that the clonal expansions of CCL-like pre-invasive lesions begin earlier during lineage evolution, and that (as expected) the rate of growth of such lesions is slower than that of less differentiated lesions or the tumor. Each AH or DCIS proliferates from an ancestral cell that has a higher mutation burden, and the ancestral cells of the invasive primary tumors begin to proliferate even later, and more quickly.

### Mixed-lineage origins of subsets of pathologically well-defined neoplasias

Two CCLs and three AHs, but none of the three DCIS, 15 IDC, or two metastatic samples, exhibited mixed-lineage origins. There may, therefore, be considerable genetic heterogeneity in some pre-invasive lesions that does not necessarily correlate with nuclear atypia or proliferation index of the sample. This suggests that genomic characterization of pre-invasive lesions would add important information to the histological characterization that is currently standard in diagnostics.

As yet unexplored mechanisms such as copy number alterations determine the variance in VAFs and so we do not yet know whether the sensitivity of our study limited the detected number of mixed-lineage clones, or whether our results are an accurate estimate of the typical number of mixed-lineage clones in pathologically well-defined samples. Due to Illumina sequencing error rates, a VAF threshold of 0.02 was required to call an SNV positive in a sample, and considering that most samples have an average of about 50% normal cell ‘contamination’, we likely missed minor subpopulations. The frequency of lineage-mixing within our set is roughly consistent with a Poisson distribution of a mean rate of about 1 in 10, as 5 out of 66 samples exhibit mixed lineage origins. This suggests that the number of ancestral lineages contributing to a pathologically defined neoplastic sample of about 1 cubic millimeter (roughly 10^6^ cells) is usually one, rarely more, at least in breast cancer.

### Recurrence of PIK3CA mutations

On the basis of the trees we were able to show repeated within-patient recurrence of two mutations, H1047R and H1047L, in PIK3CA. The mutations frequently occurred in pre-invasive neoplasias and were absent from all invasive carcinoma samples in two out of four patients. Multiple independent origins of the mutations are the best explanation for the observed patterns. Both mutations’ VAFs are measured robustly with per-patient high-quality sequence coverage over the position ranging from 87,856 reads in patient 5 to 671,239 in patient 1. The smallest number of reads in any one sample is 4,833. Across all negative samples, including all samples in patients 2 and 6 which do not have H1047R or H10147L at all, the absence of the mutations is well-supported and not due to low read coverage or another technical artifact. Rearranging the trees to force a single origin of H1047R is impossible without causing a massive conflict with all other phylogenetically informative SNVs. Alternatively to multiple independent origins one might theoretically invoke ancestral presence of the mutations and then multiple independent reversions to the normal allele. However, it is difficult to envision how a mutation that has been functionally linked to causing proliferation (for example, [26]) and that recurs so frequently in tumors (for example, [27,28]) would be selected against so as to be completely lost in a large number of samples (and billions of cells) from each patient. Also note that it is absent from the six normal and lymph control samples and present at very low frequency in only one normal sample (patient 1; Figure 3).

The VAFs of the H1047R mutation in many of the pre-invasive lesions are consistent with all cells carrying it, and therefore with it being the initial proliferative driver mutation. However, it is curious that it is absent from all IDC samples in patients 3 and 5 despite being present in several pre-invasive neoplasias in those same patients. This is consistent with previous studies that have shown PIK3CA H1047R to be highly prevalent in CCL [24,29,30]. In our prior study [29], we observed a poor correlation of CCL mutational status with the status of the accompanying carcinoma. In only 1 of 14 cases was a PIK3CA mutation present in both the patient match CCL and the concurrent carcinoma. A CCL bearing a PIK3CA mutation was typically accompanied by wild-type carcinoma, or carcinoma with a different PIK3CA mutation (in 9 of 14 cases). Given the prevalence of PIK3CA mutations in benign proliferative lesions, its absence from some IDCs even in patients that have it in other sites, and the only very slightly increased risk of cancer associated with neoplastic lesions in general and CCL in particular, it is worth considering whether PIK3CA H1047R is really a driver of neoplasia at this stage of cancer development or a comparatively benign inducer of proliferation alone.

In summary, we use phylogenetic inference to identify genetic and lineage heterogeneity in the pre-invasive compartment of breast neoplasia, including stages of hyperplasia. As this class of lesion may represent one of the earliest morphologically identifiable entities in the cancer progression pathway [16], our findings suggest that detailed genomic characterization of lesions will result in their stratification into genomically normal versus aberrant neoplasms, with clearly distinct choices for treatment and monitoring as a result. In light of the fact that genetic heterogeneity at this early stage may not manifest as a histologically detectable phenotypic difference [31], we envision two scenarios where a genomic multisample approach will be beneficial. One, if several lesions were detected by high resolution imaging in a patient, characterizing biopsies from all or a large fraction will be more representative and potentially reveal genetic heterogeneity among them. Two, if only one general site of lesion is found and a single biopsy is taken, detailed histological characterization followed by multisample analysis of histologically separated sub-lesions will also reveal the degree of heterogeneity. In either scenario, understanding the genetic and lineage heterogeneity among the samples and having the chance to identify those sites, if any, that have evolved a high mutation burden will improve monitoring and treatment for that specific patient.

## Conclusions

We used multi-sample phylogenetic inference to provide insights into the origin of driver mutations, the nature of neoplastic proliferations, and the relationship of genomically aberrant neoplasias with primary tumors. Our strategy was to use whole-genome sequencing of a limited number of samples to produce a discovery set of SNVs, and then to build highly resolved phylogenetic trees from a much-expanded sample set that did not lend itself to whole-genome sequencing, using a maximally informative subset of SNVs. The degree of genetic heterogeneity identified within the pre-invasive compartment suggests specific genomic approaches for cancer screening and early detection efforts. We show that PIK3CA driver mutations may be comparatively benign inducers of cellular proliferation.

## Additional files

Additional file 1: Table S1. Sample and data summary. Additional file 2: Figure S1. Variant allele frequencies by phylogenetic class. Additional file 3:
**Variant allele frequency tables by patient.**

## Abbreviations

AH: hyperplasia with atypia
bp: base pair
CCL: columnar cell lesion
FEA: flat epithelial atypia
DCIS: ductal carcinoma *in situ*
IDC: invasive ductal carcinoma
PCR: polymerase chain reaction
SNV: single nucleotide variant
VAF: variant allele frequency
