# Supplementary material for: Cell-lineage heterogeneity and driver mutation recurrence in pre-invasive breast neoplasia
Source: Genome Med. 2015 Apr 9;7(1):28. doi: 10.1186/s13073-015-0146-2 (PMC4410742; doi:10.1186/s13073-015-0146-2)
Supplement: Additional file 1: Table S1. — Sample and data summary. [file 13073_2015_146_MOESM1_ESM.pdf]

Table S1

Sample information	P1	P2	P3	P4	P5	P6	Totals
	**Original samples (discovery set); grey sample were not included in this study due to lack of material. Black samples were included.**						31
	Lymph			Lymph	Lymph	Lymph	
	Normal_cl	Normal	Normal				
	CCL_cl		CCL_cl	CCL_cl	CCL_cl	CCL_cl	
	CCL	CCL	CCL	CCL	CCL (2)	CCL	
	FEA					FEA	
		DCIS	DCIS			DCIS	
	IDC	IDC	IDC	IDC	IDC (2)	IDC	
	**Additional samples assayed in this study**						38
	STT6762_lym_cl	STT6714_fea	STT6735_idc	STT6756_met	STT6726_ccl/apom	STT6747_idc	
	STT6763_nl+ccl_cl	STT6716_idc	STT6736_fea	STT6757_met	STT6729_ccl	STT6748_idc	
	STT6764_fea		STT6737_ccl	STT6758_idc	STT6730_apom/pfcc	STT6749_apom	
	STT6765_ccl		STT6738_ccl	STT6759_ccl	STT6743_idc	STT6750_ccl	
	STT6766_fea		STT6739_nl	STT6760_idc	STT6744_ccl/apom	STT6751_ccl	
	STT6767_fea/adh		STT6740_ccl	STT6761_ccl	STT6745_nl	STT6752_npfcc	
	STT6768_nl		STT6741_ccl+fea			STT6753_idc	
	STT6769_fea					STT6754_nl	
						STT6755_nl	
Samples in this study (total samples)	11 (14)	6(6)	12(12)	10(10)	12(12)	15(15)	66 (69)
Neoplastic samples	7	2	7	4	7	7	34
Normal or lymph	2	1	2	1	2	3	11
DCIS or IDC	1	3	3	3	3	5	18
Other	1	0	0	2	0	0	3
Total							66
**SNV information**							
Number of reads	60,355,195	283,121,343	82,503,437	63,756,337	52,332,979	128,994,460	671,063,751
SNVs tested	130	296	192	191	180	196	1185
PCR failures	2	1	1	2	0	1	7
Negative across all samples (false positive call in original Ne	5	9	9	10	7	15	55
Germline (false negative in original Newburger 2013 study)	0	1	1	14	0	0	16
SNVs in ambiguous classes	6	6	3	1	2	1	19
SNVs used for treebuilding	117	279	178	164	171	179	1088
Total data points	1408	1770	2292	1890	2160	2925	12445
Data points used for treebuilding	1287	1674	2136	1640	2052	2685	11474
**Phylogenetic class information**							
Phylogenetically informative classes	6	6	4	4	4	6	30
Private classes	5	3	4	3	5	5	25
Total classes	11	9	8	7	9	11	55
