# Supplementary material for: Cell-lineage heterogeneity and driver mutation recurrence in pre-invasive breast neoplasia
Source: Genome Med. 2015 Apr 9;7(1):28. doi: 10.1186/s13073-015-0146-2 (PMC4410742; doi:10.1186/s13073-015-0146-2)
Supplement: Additional file 2: Figure S1. — Variant allele frequencies by phylogenetic class. [file 13073_2015_146_MOESM2_ESM.pdf]

Figure S1. Variant allele frequencies by phylogenetic class. Each point represents a single variant from a single sample, and variants from the same sample are stacked vertically. For each patient (A-F), samples with a given SNV called present are colored according to the legend and samples called absent for a given SNV are colored gray. The 0.02 VAF presence/absence cutoff used for most variants is shown as a dashed horizontal line. Variants are grouped by phylogenetic classes, indicated by a series of ones and zeros, indicating presence or absence of the mutation in the corresponding sample. Samples are in the same order as in the figure legend (and leading zeros have been omitted). For example, for Patient 1, class 10 indicates mutations present only in sample 6764_fea and class 1001 indicates mutations present in both 6767_fea-adh and 5763_IDC.

A

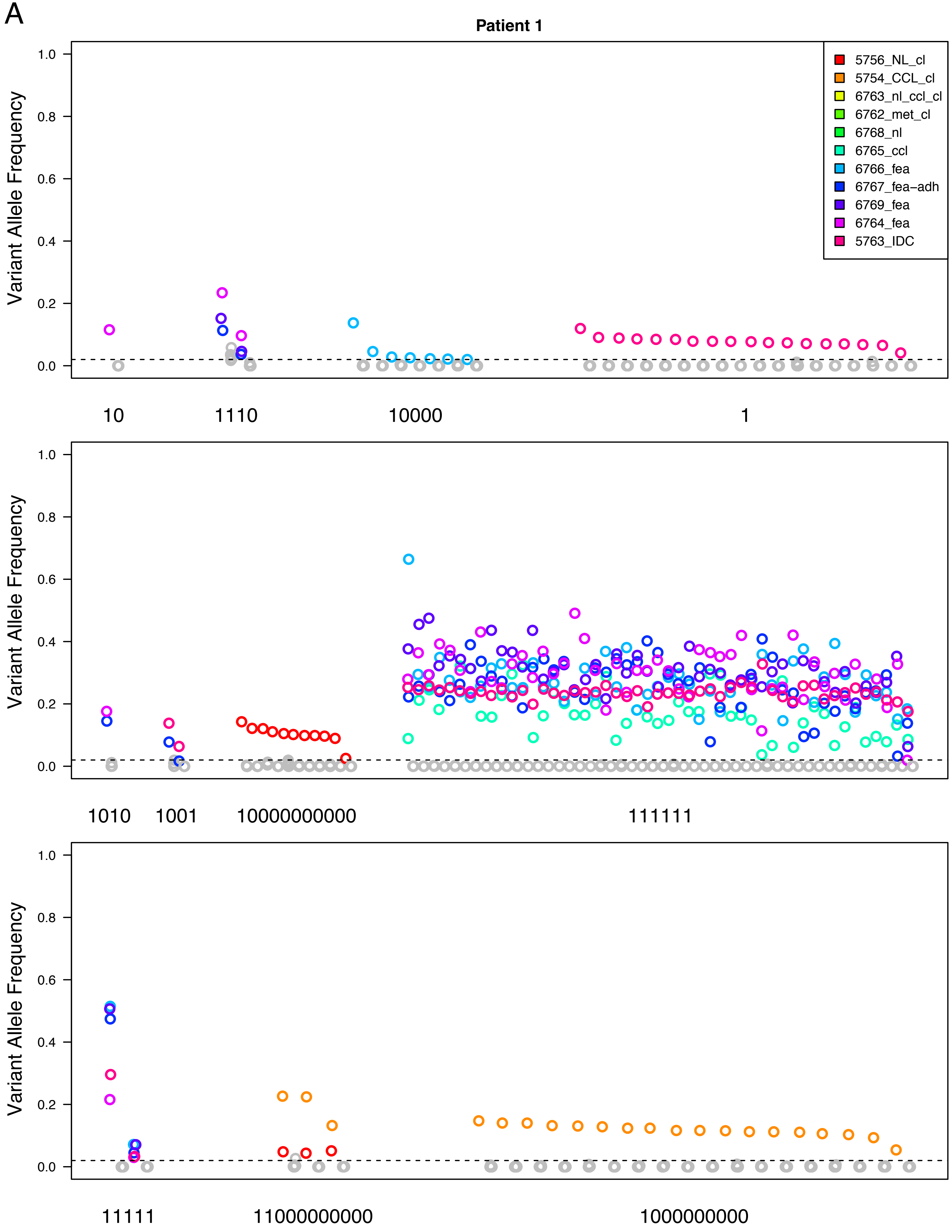
Patient 1
Variant Allele Frequency
1.0
0.8
0.6
0.4
0.2
0.0
5756_NL_cl
5754_CCL_cl
6763_nl_ccl_cl
6762_met_cl
6768_nl
6765_ccl
6766_fea
6767_fea−adh
6769_fea
6764_fea
5763_IDC
10
1110
10000
1
1010
1001
10000000000
111111
11111
11000000000
1000000000

B

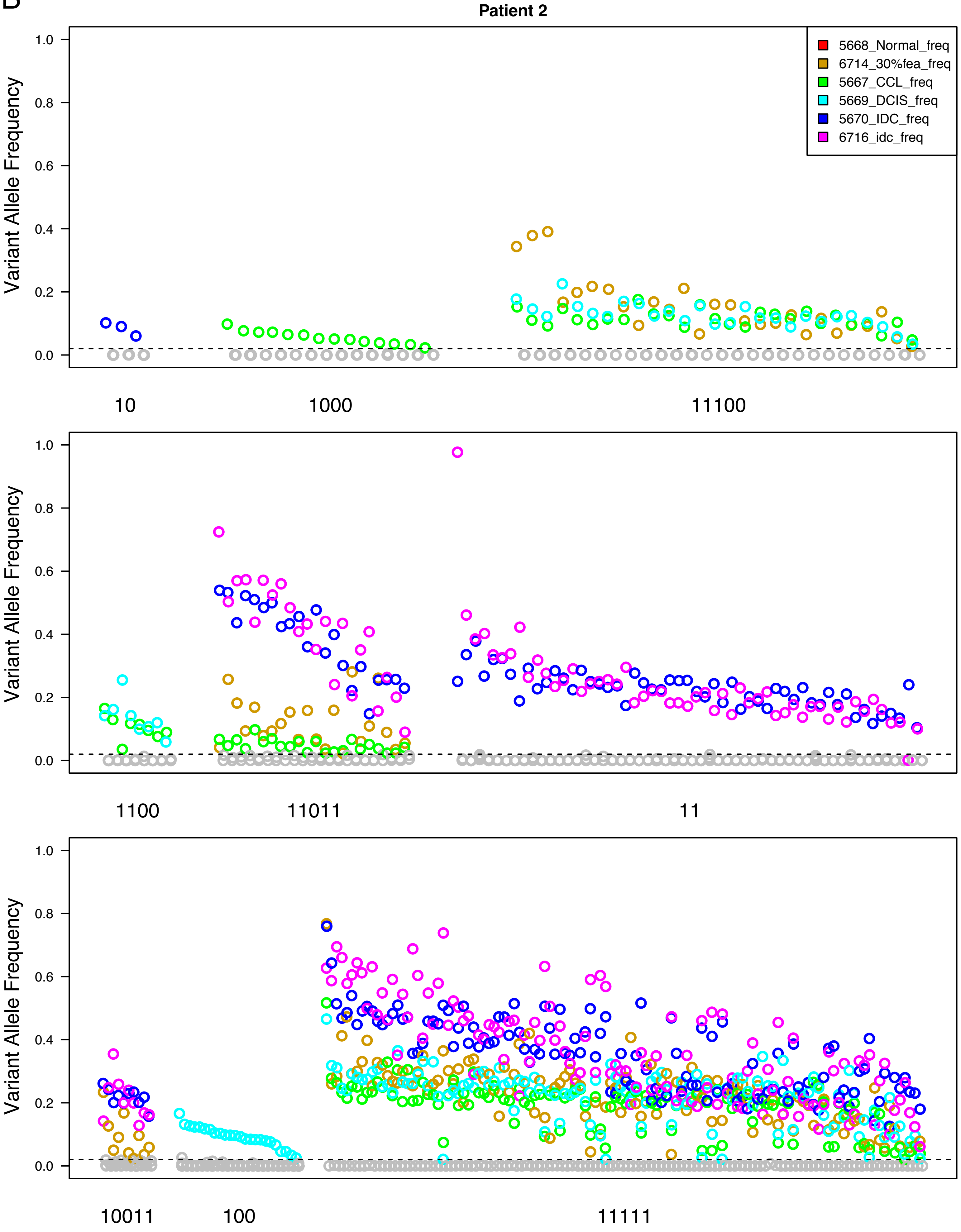
Patient 2
5668_Normal_freq
6714_30%fea_freq
5667_CCL_freq
5669_DCIS_freq
5670_IDC_freq
6716_idc_freq
Variant Allele Frequency
1.0
0.8
0.6
0.4
0.2
0.0
10
1000
11100
1100
11011
11
10011
100
11111

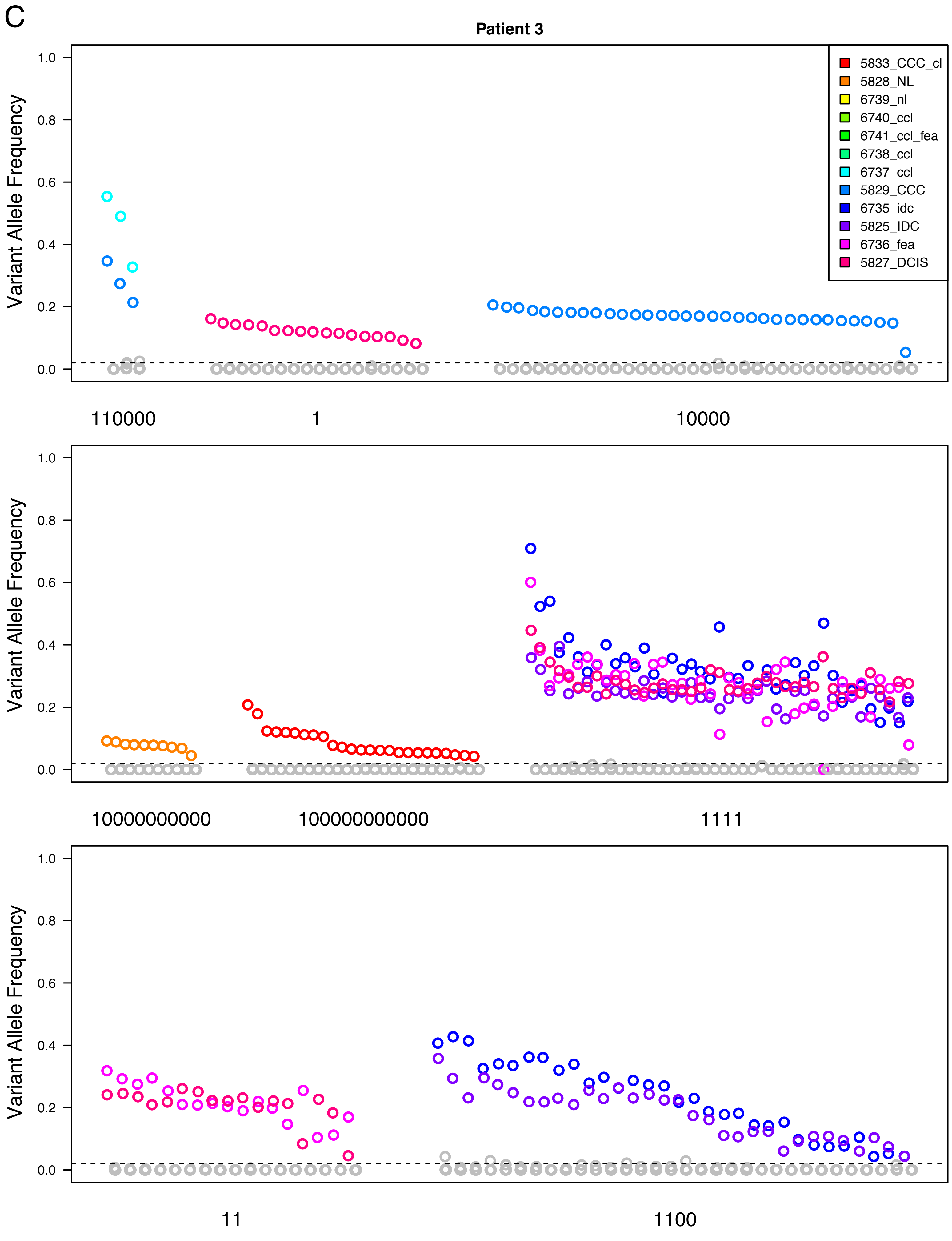
C
Patient 3
Variant Allele Frequency
1.0
0.8
0.6
0.4
0.2
0.0
5833_CCC_cl
5828_NL
6739_nl
6740_ccl
6741_ccl_fea
6738_ccl
6737_ccl
5829_CCC
6735_idc
5825_IDC
6736_fea
5827_DCIS
110000
1
10000
10000000000
100000000000
1111
11
1100

D

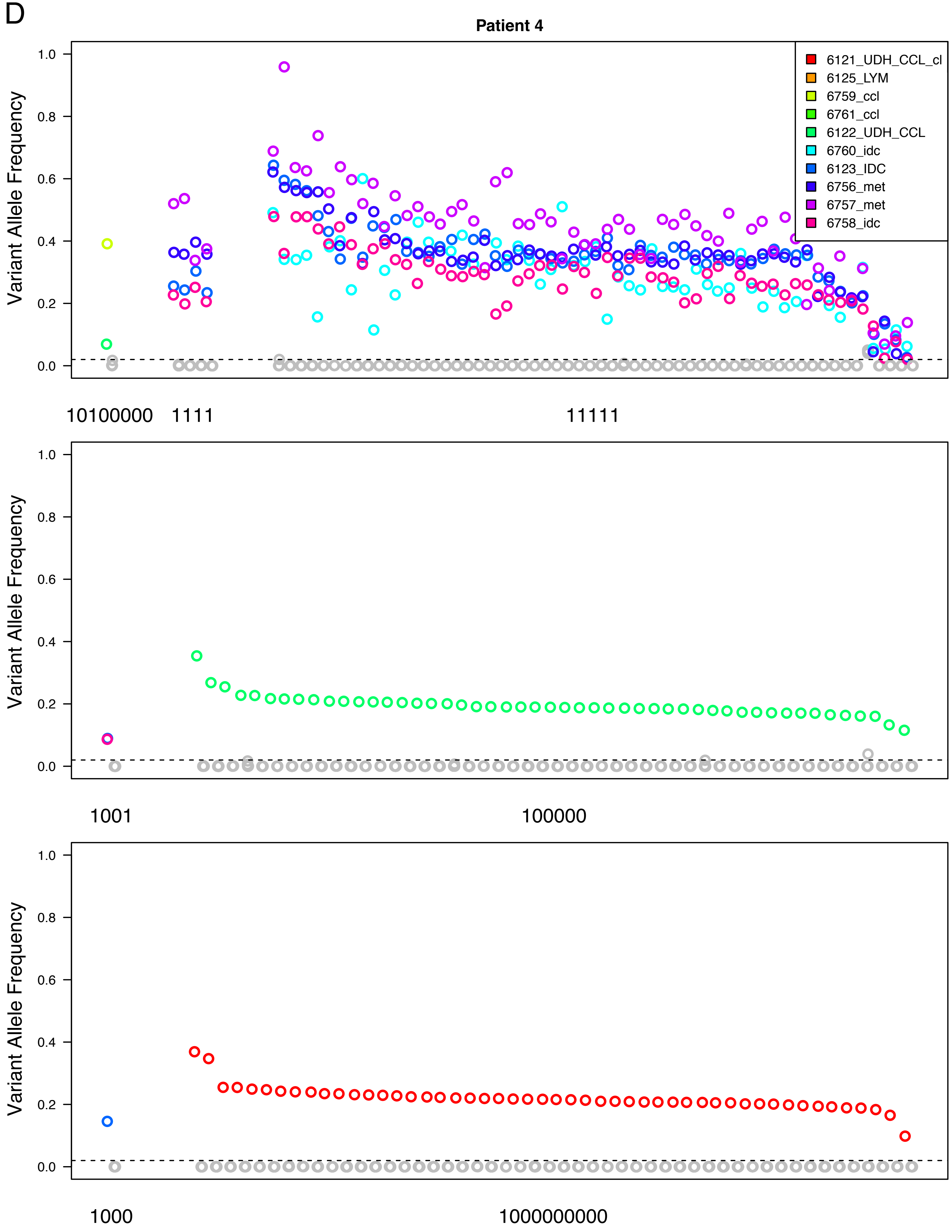

Patient 4
6121_UDH_CCL_cl
6125_LYM
6759_ccl
6761_ccl
6122_UDH_CCL
6760_idc
6123_IDC
6756_met
6757_met
6758_idc
Variant Allele Frequency
1.0
0.8
0.6
0.4
0.2
0.0
10100000
1111
11111
1001
100000
1000
1000000000

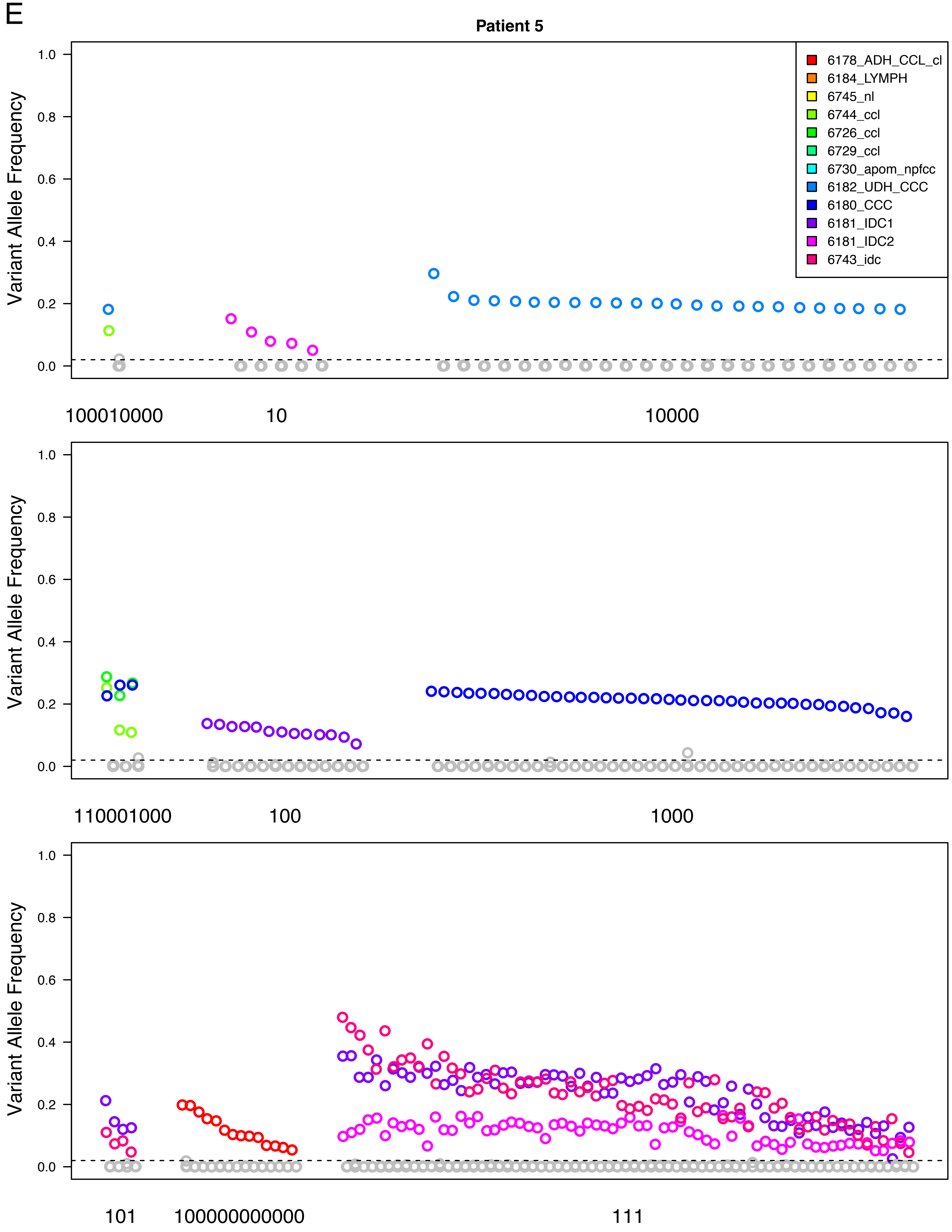
E
Patient 5
6178_ADH_CCL_cl
6184_LYMPH
6745_nl
6744_ccl
6726_ccl
6729_ccl
6730_apom_npfcc
6182_UDH_CCC
6180_CCC
6181_IDC1
6181_IDC2
6743_idc
Variant Allele Frequency
1.0
0.8
0.6
0.4
0.2
0.0
100010000
10
10000
110001000
100
1000
101
100000000000
111

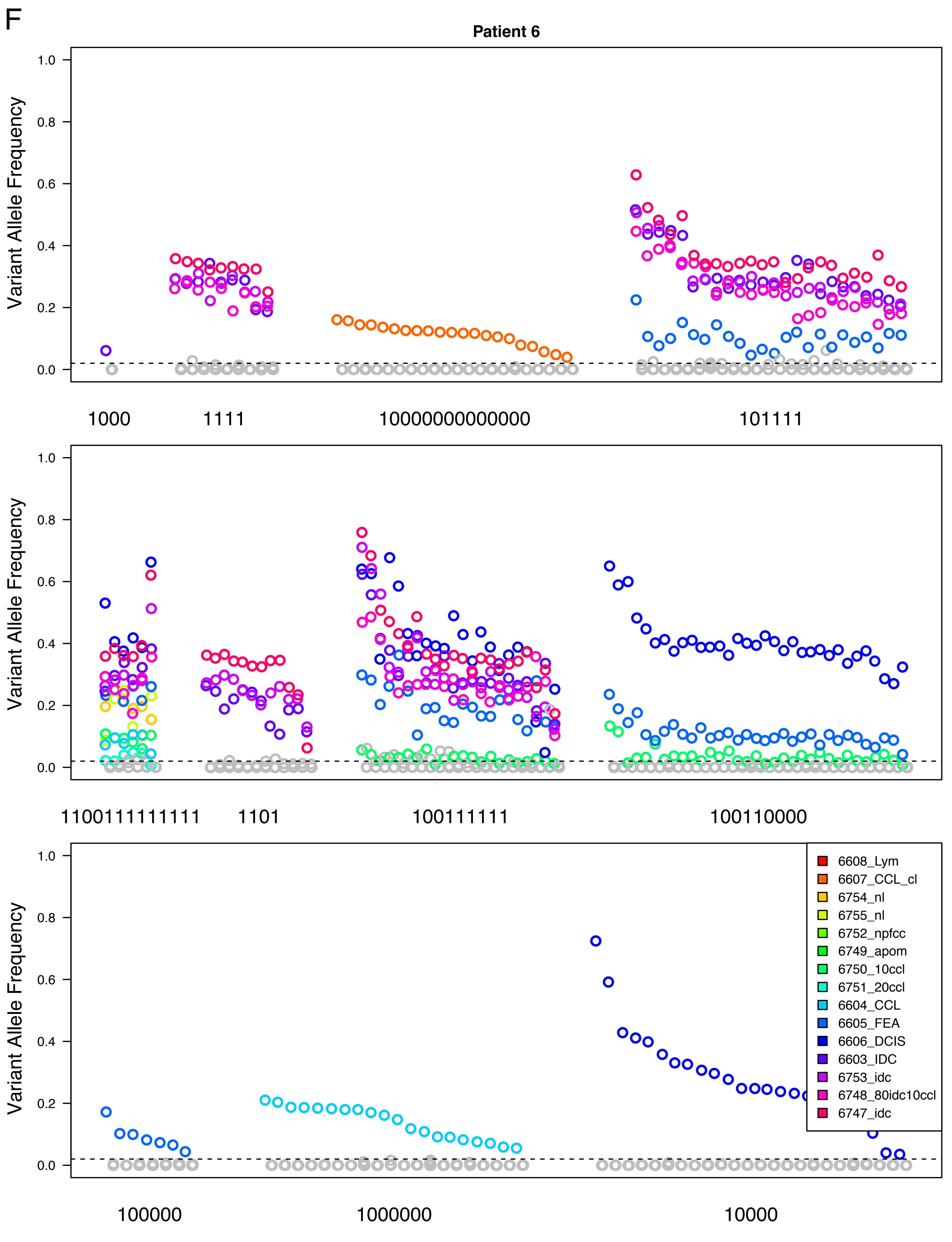
F
Patient 6
Variant Allele Frequency
1000
1111
10000000000000
101111
1100111111111
1101
100111111
100110000
100000
1000000
10000
6608_Lym
6607_CCL_cl
6754_nl
6755_nl
6752_npfcc
6749_apom
6750_10ccl
6751_20ccl
6604_CCL
6605_FEA
6606_DCIS
6603_IDC
6753_idc
6748_80idc10ccl
6747_idc
